# Temperature-Dependent Superplasticity and Strengthening in CoNiCrFeMn High Entropy Alloy Nanowires Using Atomistic Simulations

**DOI:** 10.3390/nano11082111

**Published:** 2021-08-19

**Authors:** Pawan Kumar Tripathi, Yu-Chen Chiu, Somnath Bhowmick, Yu-Chieh Lo

**Affiliations:** 1 International College of Semiconductor Technology, National Yang Ming Chiao Tung University, 1001 University Road, Hsinchu 300, Taiwan; pawantripathi19@gmail.com; 2 Department of Materials Science and Engineering, Indian Institute of Technology Kanpur, Kanpur 208016, India; bsomnath@iitk.ac.in; 3 Department of Materials Science and Engineering, National Yang Ming Chiao Tung University, 1001 University Road, Hsinchu 300, Taiwan; wade890093@gmail.com

**Keywords:** superplasticity, nanowires, high entropy alloys, TWIP, TRIP

## Abstract

High strength and ductility, often mutually exclusive properties of a structural material, are also responsible for damage tolerance. At low temperatures, due to high surface energy, single element metallic nanowires such as Ag usually transform into a more preferred phase via nucleation and propagation of partial dislocation through the nanowire, enabling superplasticity. In high entropy alloy (HEA) CoNiCrFeMn nanowires, the motion of the partial dislocation is hindered by the friction due to difference in the lattice parameter of the constituent atoms which is responsible for the hardening and lowering the ductility. In this study, we have examined the temperature-dependent superplasticity of single component Ag and multicomponent CoNiCrFeMn HEA nanowires using molecular dynamics simulations. The results demonstrate that Ag nanowires exhibit apparent temperature-dependent superplasticity at cryogenic temperature due to (110) to (100) cross-section reorientation behavior. Interestingly, HEA nanowires can perform exceptional strength-ductility trade-offs at cryogenic temperatures. Even at high temperatures, HEA nanowires can still maintain good flow stress and ductility prior to failure. Mechanical properties of HEA nanowires are better than Ag nanowires due to synergistic interactions of deformation twinning, FCC-HCP phase transformation, and the special reorientation of the cross-section. Further examination reveals that simultaneous activation of twining induced plasticity and transformation induced plasticity are responsible for the plasticity at different stages and temperatures. These findings could be very useful for designing nanowires at different temperatures with high stability and superior mechanical properties in the semiconductor industry.

## 1. Introduction

Nanomaterials are new age materials exhibiting exceptional mechanical, electronic, and optical properties due to their unique structure and high surface-area to volume ratio [1]. Among nanomaterials, one dimensional (1D) metallic nanowires (NWs) are widely used for different applications including nanoelectronics, optoelectronics, energy harvesting and storage, ultrasensitive sensing, and nanoelectromechanical devices [2,3,4]. At the nanoscale, 1D NWs usually exhibit ultra high strength, differently from bulk materials, which makes them ideal candidates for studying their fundamental deformation mechanisms. Dislocation nucleation from free surfaces and its subsequent propagation through NWs has been found to be a dominant deformation mechanism in metallic NWs such as Au, Ag, Cu, and Ni [5,6,7,8,9,10,11,12,13]. This enables reorientation to a more preferred phase, resulting in exceptional mechanical properties such as superplasticity, high yielding strength, pseudoelasticity, and shape memory effects [5,6,7,8,9].

High entropy alloys (HEAs) [14] have gained much attention recently due to their unique solid solution hardening combined with high ultimate tensile strengths, uniform elongation, high cryogenic fracture toughness, and excellent ductility if consisting of a single phase face-centered cubic (fcc) with a large number of slip systems [14,15,16]. CoNiCrFeMn alloy is such an equiatomic HEA with a single phase fcc structure firstly reported by Cantor et al. [17]. Although, the elements in this alloy possess different crystal structures, it crystallizes as a solid solution of single phase fcc. There are numerous experimental [15,18,19,20,21,22] and simulation [23,24,25,26] studies reporting the mechanical properties of CoNiCrFeMn alloy. Otto et al. [18] reported an increment in the yield strength, ultimate tensile strength and elongation to fracture with decreasing temperature especially in the cryogenic range. Surprisingly, ductility was also improved significantly at low temperatures with strength. Similar behavior was observed in the studies of Gludovatz et al. [15]. The tensile strength increased from 730 to 1280 MPa as temperature is lowered from ambient temperature (293 K) to cryogenic temperature (77 K). Xiao et al. [23] inflicted tensile deformation on CoNiCrFeMn HEA NWs using MD simulations at room temperature and reported superplasticity and improved strengthening in single crystal HEA NWs due to the mechanism being dominated by the FCC to HCP martensitic transformation. Shape memory effects are also found under certain conditions when only one single slip is activated.

Gali et al. [27] reported a strong temperature-dependent decrease in yield strength with an increase in temperature from 77 to 1273 K with relatively weak strain rate dependence. Elongation to fracture was increased by 1.5 times to greater than 60% when temperature decreased from room temperature to cryogenic temperature. At 77 K, onset of necking was delayed due to deformation induced nanotwinning with a high degree of work hardening resulting in increased ductility. Woo et al. [28] studied high temperature deformation behavior of CoNiCrFeMn at 800 and 1000 K using in situ neutron diffraction and reported dominant deformation mode is dislocation glide at 800 K and diffusion controlled dislocation creep at 1000 K. At 800 K, dislocation cell structure and occasional occurrence of nanotwins are observed at 1.7% strain in the early stages of deformation. On the other hand, at 1000 K, wavy and zigzag dislocations indicate crossing of thermal energy barrier for dislocation creep. Yeh et al. [29] have further concluded that severe distortion in the high entropy alloys can create much more resistance than traditional alloys at the same temperature. During tensile deformation of HEAs, it has also been reported that HEAs show serration characteristics on flow stress curves similar to the many functional and structural materials, intermetallics, Al-Mg alloys, shape-memory alloys, twinning induced plasticity (TWIP) and transformation induced plasticity (TRIP) steels [18,28,30,31]. Serration behavior can be characterized as a row of sharp or tooth-like projections on flow stress curves of solid materials caused by dislocation interactions [30,31].

Strain hardening in conventional metals is generally described with Taylor’s relation where an increase in flow stress is related to the increased dislocation densities [32]. Deng et al. [33] performed an MD simulation on periodically twinned FCC NWs and found a fundamental transition of plasticity from a sharp yield point and strain softening to significant strain hardening as stacking fault energy (SFE) of the metal decreases. It was established that plastic behavior of metallic NWs mainly depends upon process of nucleation and propagation of dislocations from free surfaces and their interactions with pre-existing defects. A fundamental transition of plasticity in twinned metal NWs from sharp yield and strain-softening to significant strain hardening occurs when the unstable stacking fault energy $\left(\gamma_{USF}\right)$ of the metal decreases.

Nowadays, HEA nanoparticles are synthesized successfully and are able to exhibit better catalytic performances [34]. The successful synthesis of HEA nanowires is expected very soon. Therefore, understanding the origin of such excellent properties is an important scientific issue for the design and application of HEA NWs. The partial dislocation movement is responsible for high elongation and ductility of NWs, while FCC-HCP martensitic transformation contributes to the strain hardening behavior [21]. There can be four possible scenarios in FCC HEAs: the nucleation of a single leading partial dislocation, the nucleation of a full dislocation with leading and trailing partial dislocations, deformation twinning, and FCC to HCP martensitic transformation [21,35]. Stacking fault energy (SFE) is found to play an important role in activation of partial dislocations, and also its further propagation. Therefore, we have chosen silver (Ag) nanowires which have similar SFEs to the CoNiCrFeMn HEAs. Further, it has also been reported that the nucleation of dislocation and twinning is often dependent on stacking fault energy in the metals. Molecular dynamics simulations are a well known technique to investigate the atomic mechanisms related to mechanical properties [5,6,7,8,9,36], phase transformation [37,38], deformation behavior [5,6,23,39,40], etc. of both the bulk and nanoscale systems. Therefore, in the current study, we have performed MD simulations on both single element Ag NWs and multicomponent CoNiCrFeMn HEA NWs to investigate their deformation behaviors as they have almost similar stacking fault energies. The multicomponent HEA alloy consists of Co, Cr, Fe, Mn, and Ni each in 20 at. %.

## 2. Simulation Details

### 2.1. Interatomic Potential

An embedded atom method (EAM) potential developed by Williams and Mishin [41] is used to mimic the interaction among the Ag atoms. This version of potential has been successfully employed to reproduce the lattice parameter, cohesive energy, elastic constants, thermal expansion coefficients, lattice defect energies, phonon frequencies, and energies of the alternate structures of Ag. Apart from this, this potential is used in many deformation studies related to Ag [42,43,44].

To describe the interaction among the atoms of CoNiCrFeMn HEA alloy NWs, a second nearest neighbor modified embedded atom method (2NN MEAM) potential developed by Choi et al. [45] is used in this study. This potential can accurately describe the properties related to plastic deformation, phase transformation, sluggish diffusion, and solid-solution strengthening and has also been used in numerous studies [23,24,25,45].

### 2.2. Nanowire Simulation Details

**Figure 1:**
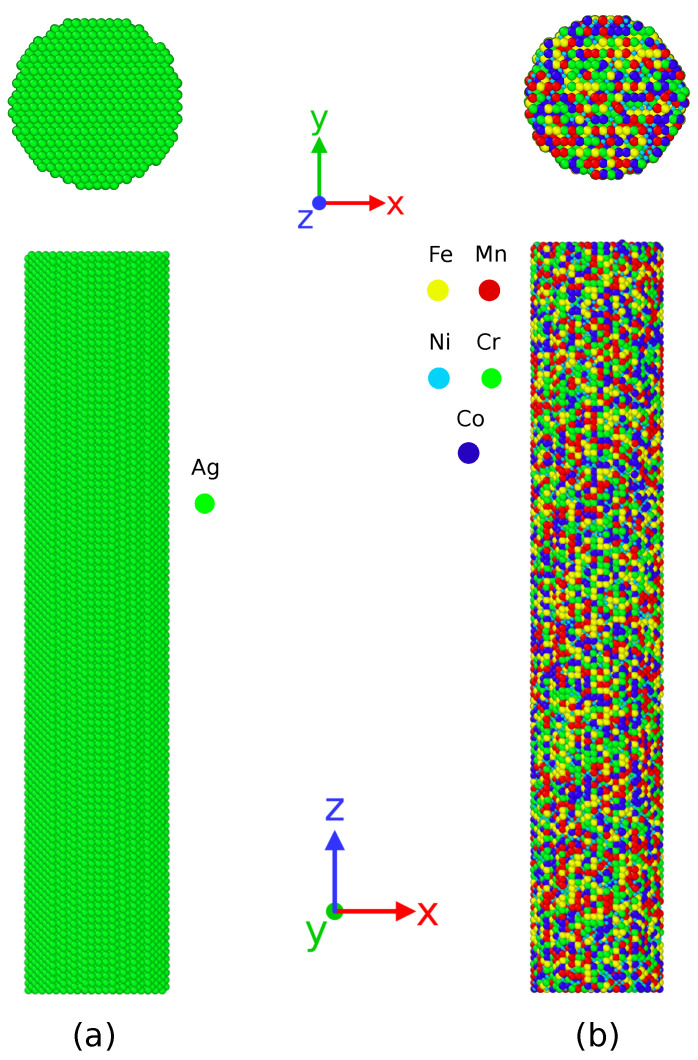
Cylindrical nanowire and corresponding cross-section of (**a**) silver (Ag) nanowire (**b**) CoNiCrFeMn HEA nanowire. The elements present in HEA nanowire are shown in different colors.

To investigate the temperature dependent deformation mechanism during the tensile deformation of CoNiCrFeMn HEA and Ag NWs, we have conducted a series of atomistic simulations at 77, 300, and 1000 K. Molecular dynamics simulations as implemented in a large-scale atomistic/molecular massively parallel simulator (LAMMPS) [46] are performed for each NW at a constant strain rate. The Ag and HEA NWs with a circular cross-section of (110) are shown in Figure 1 a and c are extracted from a bulk FCC crystal along the [110] orientation. A rectangular simulation box with z direction as the deformation direction is created to simulate tensile behavior. The Ag NW consists 47,250 atoms while HEA NW contains 55,216 atoms. The x, y, and z axes of the simulation box are oriented along $\left[\bar{1}10\right]$, [001], and [110] directions respectively. The NW occupies the center of the simulation box with periodic boundary conditions along the z axis and free boundary conditions in the other two directions. Other crystallographic details can be seen in Table 1.

Before we begin the simulation, all the nanowires are first relaxed using conjugate gradient minimization. Subsequently, all the nanowires are equilibrated at their respective temperatures and zero pressure using Langevin thermostat and Berendsen barostat, respectively, in the z-direction. Subsequently, a microcanonical or NVE (number of particles, volume, and energy are conserved) ensemble is used to relax the nanowire for 50 ps. The simulation box remains periodic in all three dimensions and the time step for the integration is taken as 1.0 fs. After equilibration, a constant strain rate of $10^{8}$ 1/s is applied along the z direction. During tensile deformation, a canonical or NVT (temperature, volume, and energy are conserved) ensemble is used to maintain temperature in a constant volume environment. The atomic stress of each atom is calculated using the Virial theorem [47]. The tensile stress in the z-direction is defined by averaging the per atom axial stress over all the atoms. In order to identify the defects nucleated during tensile deformation, the common neighbor analysis as implemented in OVITO [48] is used. Atoms in green, red, and grey color represent fcc, hcp, and other atoms, respectively. The twin boundary can be identified as a single layer of red atoms.

## 3. Results and Discussion

### 3.1. Deformation Behavior of Ag NWs and CoNiCrFeMn HEA NWs

**Figure 2:**
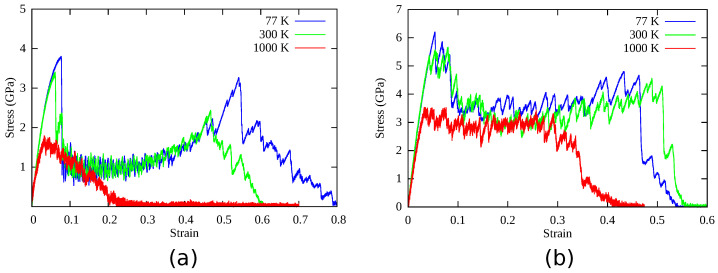
Stress–strain curves obtained at different temperatures during uni-axial tensile deformation of (**a**) Ag nanowires and (**b**) HEA nanowires.

**Figure 3:**
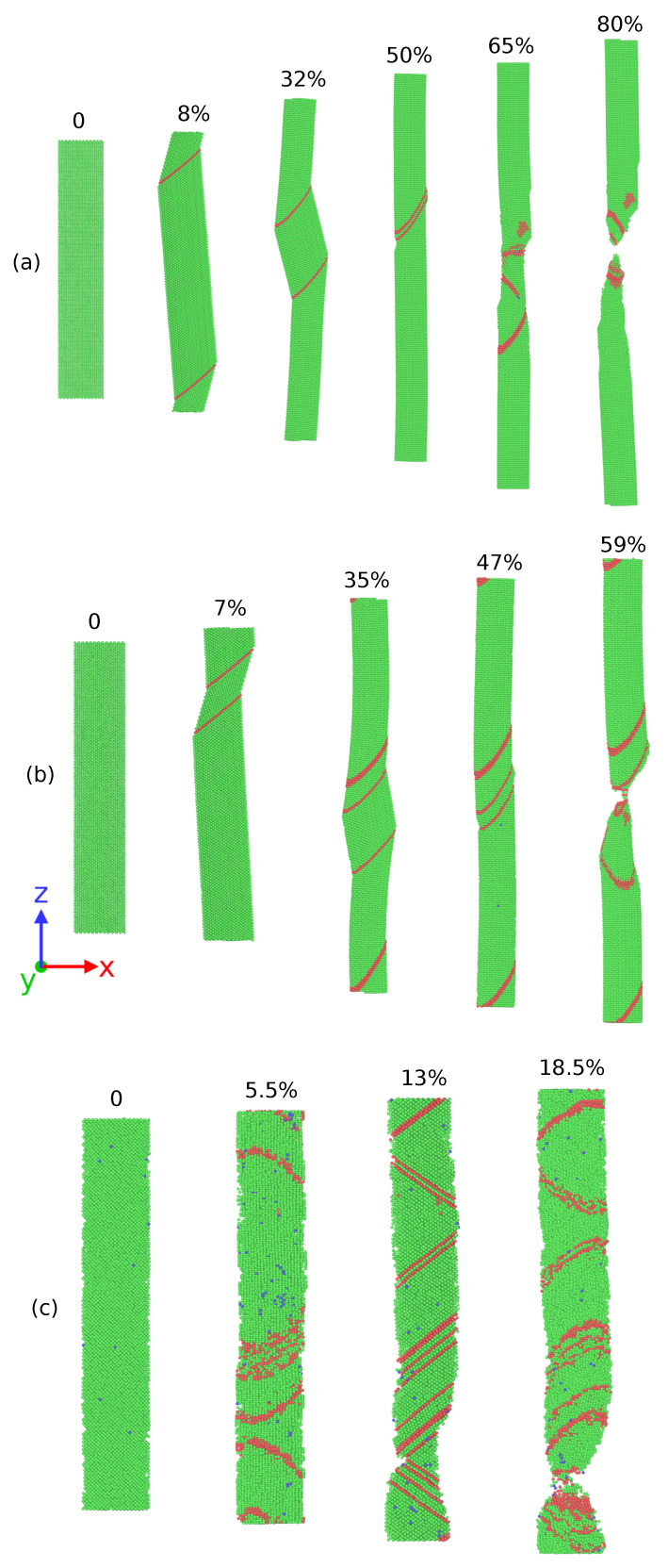
Atomic snapshots showing deformation behavior of single crystal Ag nanowires at (**a**) 77 K; the area between two twin boundaries transforms from (110) to (100) up to 50% strain value. Subsequently, single crystal nanowire reoriented along (100) deforms during 50-80% strain. (**b**) 300 K, complete reorientation from (110) to (100) single crystal could not take place. (**c**) 1000 K, no twinning induced reorientation observed. The percentage indicates the value of the applied uniaxial strain in the Z direction. The green and red atoms indicate FCC and HCP atoms respectively.

**Figure 4:**
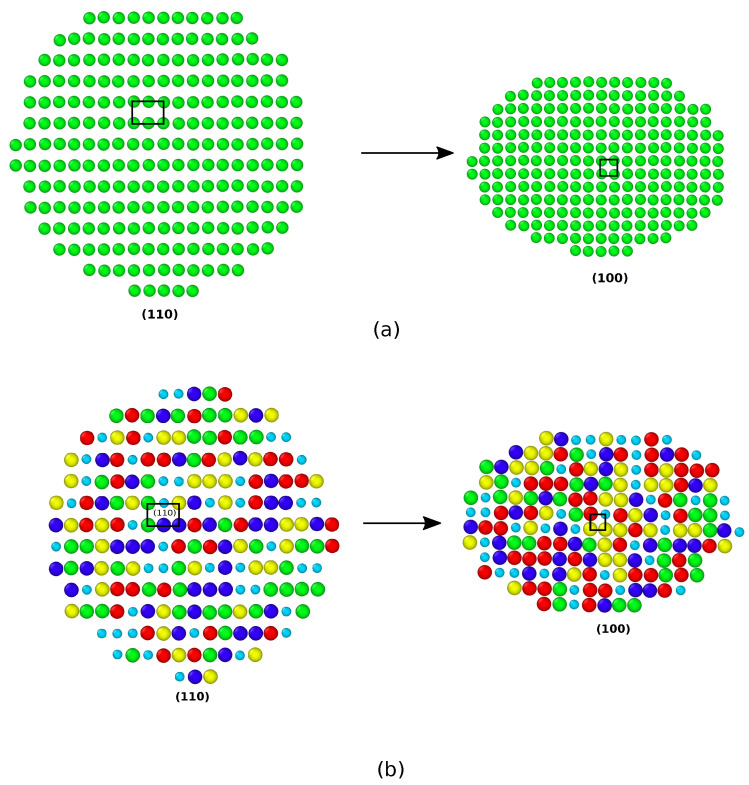
Atomic snapshots of the NW cross-sections at 77 K before and after re-orientation from (110) to (100) phase during tensile deformation of (**a**) silver (Ag) nanowire and (**b**) CoNiFeMnCr HEA nanowire. In CoNiFeMnCr NW, there is no complete re-orientation of the nanowire.

Figure 2 shows the stress–strain curves of the Ag NWs and CoNiCrFeMn HEA NWs at 77, 300, and 1000 K respectively. At 77 K, Ag NW deforms via nucleation and propagation of a partial dislocation from the free surface, leaving a stacking fault inside the NW (see Figure 3a). As deformation continues, the stacking fault further separates into two twin boundaries, which extend and continue to grow through entire NW. The cross-section area of the NW eventually converts from (110) to (100), resulting in two peaks in the SS curve, as shown in Figure 2a. The area between two twin boundaries is transformed as also shown in Figure 3a between 8% to 32% elongation in the Ag NW. The phase transformation is mainly driven by the surface stresses which enable the reduction in surface energy in the nanowire. On further loading, the difference between two boundaries decreases and finally vanishes at around 50% elongation, resulting in an improved work hardening. As shown in Figure 2a, the two peak stresses at 77 K indicate the yielding behaviors of the respective phases. Thus, the re-orientation of the Ag NW from (110) into a more preferred (100) (see Figure 4) phase, as also shown in Figure 3a, conferred additional plasticity to the Ag NW. This behavior is reported in numerous studies [7,8,9,10] related to deformation in metallic nanowires. At elevated temperatures, the propagation of twin boundaries is retarded due to thermal vibrations and complete transformation from (110) to (100) phase is not possible. Eventually, nanowire fails by the necking as the multiple glide mechanism activated at 300 K as shown in Figure 3b. In contrast to this, at 1000 K, there is no re-orientation at the cross-section and nanowire fails due to the necking in the region of partial dislocation junctions due to activation of partial dislocations on different slip planes resulting in a lower elongation to failure (see Figure 3c). Thus, at low temperatures (i.e., at 77 and 300 K), twinning induced plasticity remains an operative mechanism for the failure of silver (Ag) nanowires, while at high temperatures (1000 K), dislocation slip dominates as a possible deformation mechanism.

**Figure 5:**
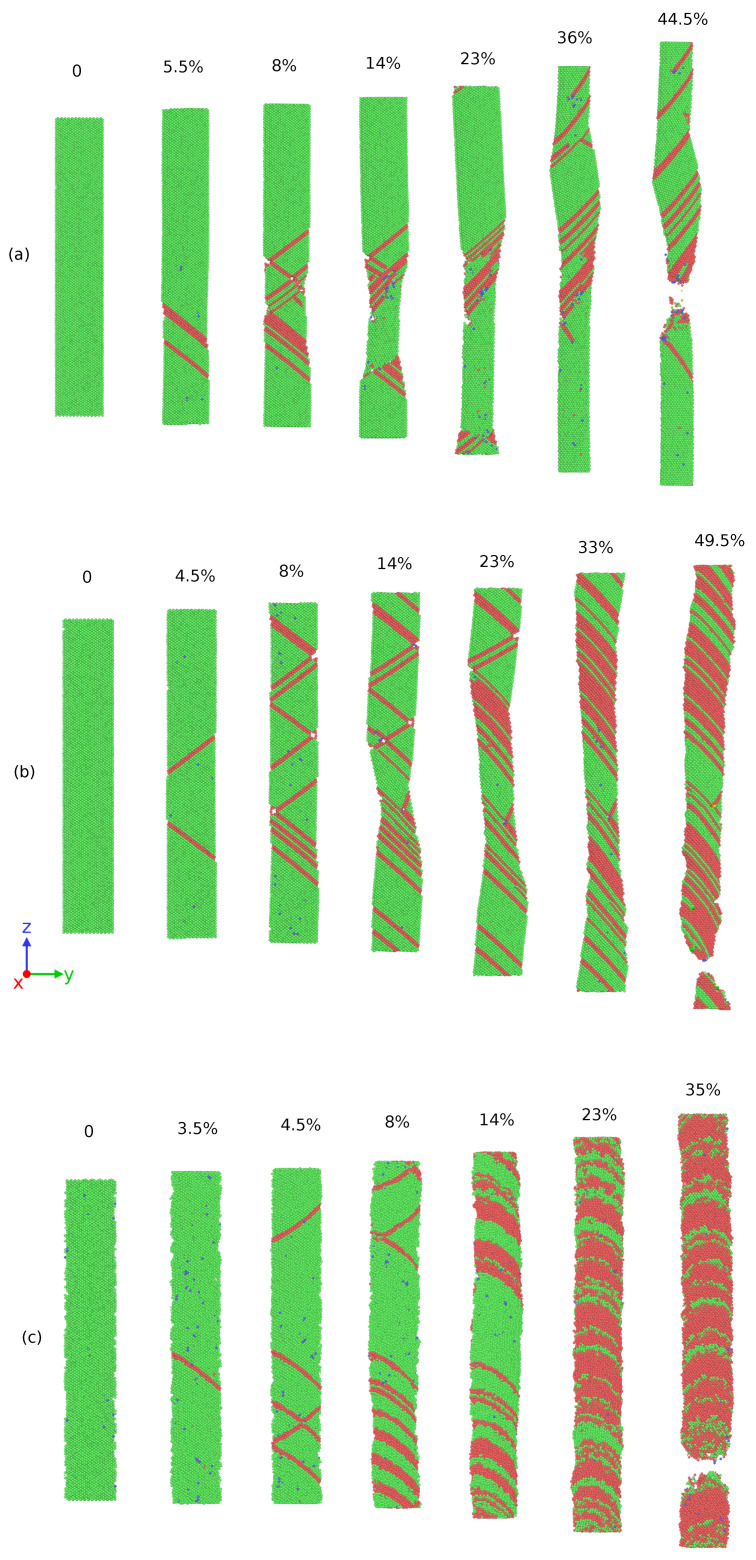
Atomic snapshots showing deformation behavior of single crystal HEA nanowires at (**a**) 77 K, reorientation from (110) to (100) phase begins after nucleation and glide of twin boundaries at 8% strain onwards. Complicated dislocation interaction is visible during deformation (see also animation S1 and S2 in the supplementary information); (**b**) 300 K; and (**c**) 1000 K. The percentage indicates the value of applied uniaxial strain in the Z direction.

**Figure 6:**
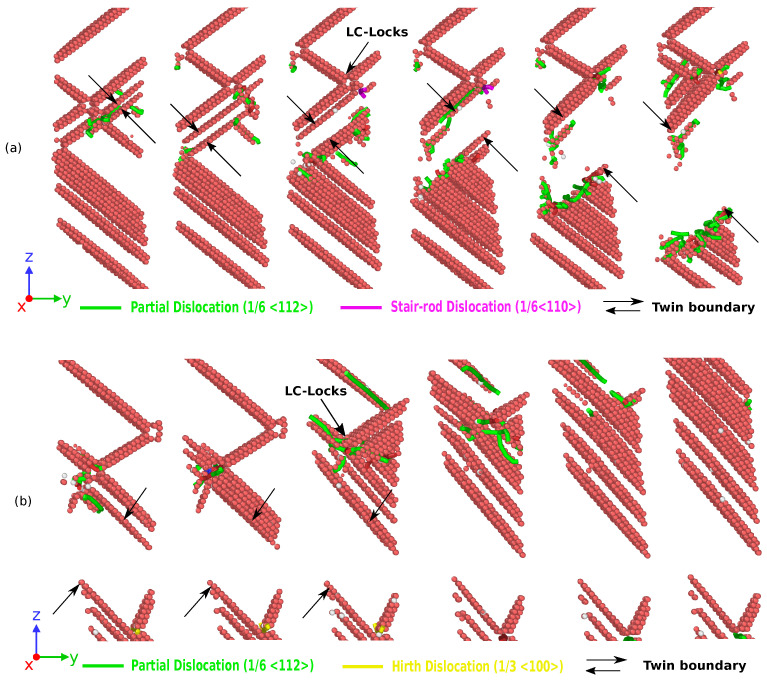
Dislocation interaction and formation of twin boundary from an intrinsic stacking fault during tensile deformation of CoNiCrFeMn NWs. This further results in formation of Lomer Cottrell locks and stair-rod dislocations. The region between two black arrows shows glide of twin boundary, transforming it from (110) to (100). The green, pink, and yellow wire shapes represent partial dislocations, stair-rod dislocations, and Hirth dislocations respectively. Dislocation analysis is carried out using the DXA algorithm as implemented in OVITO [48,50]. (**a**) 77 K; (**b**) 300 K.

On the other hand, in CoNiCrFeMn HEA NW, stress increases monotonically with strain at 77 K, as shown in Figure 2b, and reaches its peak value around 6.2 GPa at 5–6% elongation, which looks similar to the first yield point in Ag NW stress–strain curves shown in Figure 2a. The abrupt change is due to yielding, showing the nucleation of a partial dislocation in both HEA and Ag single crystal nanowires (see Figure 3a and Figure 5a), respectively. After a succession of such events in HEA NW, a remarkable drop in stress indicates the formation of deformation twins (see also Figure 6). The flow stress curve also shows serration behavior as stress drops and strain increases which is also reported in previous studies [18,28]. The serration behavior is caused by the interaction of moving dislocations which further results in formation of Lomer Cottrell locks [21,49]. Upon further loading, nucleation of partial dislocations continues even after twin formation as it can be identified with further rise and drops in the SS curve as shown in Figure 2b. In contrast to Ag NW, the movement of twin boundary through the axis of nanowire is somehow limited by stacking fault networks which enables additional work hardening in the HEA NW. Subsequently, accumulation of dislocations and stacking faults result in a phase transformation to the HCP phase, also known as martensitic transformation responsible for additional ductility. Simultaneous activation of twinning and martensitic transformation delays the onset of necking which results in a higher elongation to the fracture. Thus, the operative deformation mechanism is dominated by twinning induced plasticity with martensitic transformation resulting in exceptional ductility of HEA NW at 77 K. Similar to Ag NW, a partial re-orientation from (110) to (100) as shown in Figure 3a and Figure 4a induced by nucleation and propagation of twin boundary also takes place in CoNiCrFeMn HEA NW (see Figure 4b and Figure 5a).

At 300 K, stress in CoNiCrFeMn HEA NW reaches up to 5.5 GPa at around 5% strain, as shown in Figure 2b during elastic deformation. Multiple partial dislocations nucleate simultaneously at different slip planes across NW as can be seen in Figure 5b. It has been thought that HEAs own the lattice distortion effect which will be amplified under higher temperatures. Therefore, the activation barriers of dislocation nucleation could be lower than those at 77 K, which gives rise to more partial dislocation nucleation at different sites. Subsequently, on further loading, more partial dislocations nucleate and glide through the NW, inducing the deformation twinning and martensitic transformation to the HCP phase. Interestingly, the reorientation mechanism can still exist and contribute to the extended plasticity until the fracture of NW caused by necking as shown in Figure 5b. Overall, the exceptional strength-ductility trade-off of HEA NW at low temperature is a synergistic result of operative mechanisms involving the twinning, phase transformation, and also the re-orientation of the NW cross-section.

At 1000 K, owing to the thermal effect raising the nucleation frequency, the yielding occurs earlier with a value of only half of that at low temperature. Unlike the SS curve of Ag at 1000 K where the flow stress and ductility decay quickly to fulfil the failure, the flow stress of HEA NW can still maintain at a certain degree as good as the flow stress at the low temperature. The good refractory property of the HEA NW in this work is attributed to the same synergistic mechanism as the aforementioned one. The difference is that the phase transformation induced plasticity seems to be more dominant at high temperatures so that the fraction of the HCP phase is much more than the low-temperature cases. We also find that the increasing fraction of the HCP phase is associated with the unlock behavior of the Lomer Cottrell locks. Due to the interaction of leading and trailing partial dislocations, Lomer Cottrell locks also form at 1000 K similar to the cases at low temperature. However, the Lomer Cottrell locks do not become impediments to the following dislocation motion but can be unlocked due to transformation into the HCP phase enabled by accumulation of stacking faults at the Lomer Cottrell locks. With this unique mechanism, the stacking faults at different orientations gradually quit the NW, and eventually, the stacking faults at the same orientation are left behind, as can be seen in Figure 5b,c. Therefore, as the loading strain increases, the fraction of the HCP phase also increases quickly through the aforementioned mechanism. Since the HCP phase has fewer slip systems, the HCP phase sections will provide the secondary reinforcement for the NW. Later, the necking occurs at one of the HCP phase sections in the NW.

**Figure 7:**
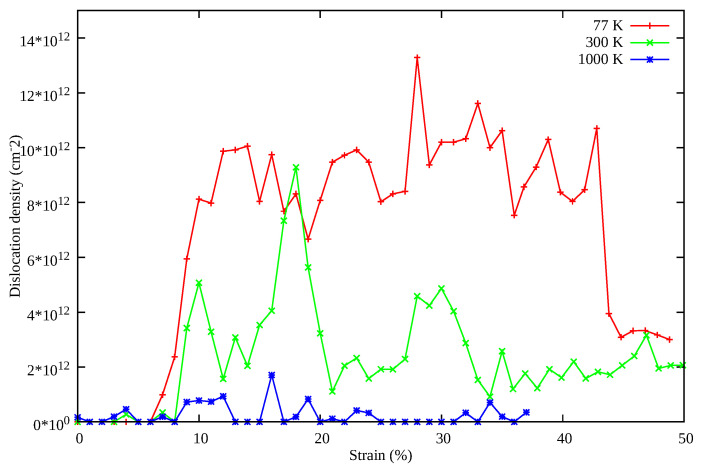
Dislocation density at different temperatures during uni-axial tensile deformation of CoNiCrFeMn HEA NWs.

Work hardening is often seen in metallic systems, leading to better ductility. Lomer Cottrell locks enable a complex dislocation network and provide a strong resistance to the propagation of moving dislocations and are inherently responsible for the work hardening behavior. In our simulations at low temperature where dislocation density is high as shown in Figure 7, Lomer Cottrell locks form a strong barrier to the moving twin boundary shown by black arrows in Figure 6a. Consequently, this creates a high dislocation interaction activity at the location where the twin boundary propagates and interacts with Lomer Cottrell locks. This in turn retards the motion of propagating twin boundaries and also prevents the complete transformation of NW in contrast to Ag NW at 77 K. However, the area between two twin boundaries is transformed from (110) to (100) as shown in Figure 4. Thus, multiple synergistic mechanisms are activated, beginning from partial dislocation nucleation, and twinning induced plasticity overtakes at around 8% strain. At a later stage, martensitic transformation also activates, delaying the onset of necking to failure around 45% strain. The synergistic effect of these mechanisms provides exceptional strength, ductility, increased work hardening rate and damage tolerance to the HEA NW. The above properties are also observed in the existing experimental works [18,21,51].

### 3.2. Strengthening Mechanism of CoNiCrFeMn HEA NWs

The tensile deformation in CoNiCrFeMn HEA NWs is primarily proceeded by nucleation and glide of 1/6<112> type Shockley partial dislocation loops which further creates extended stacking faults as shown in Figure 5 in the NW identified as HCP atoms. The glide of first Shockley partial creates an intrinsic stacking fault, which is followed by the second and third Shockley partials on adjacent slip planes.

Strengthening of the nanowires is dependent on the nature of dislocation interaction at different temperatures. On two different (111) and $\left(\bar{1}11\right)$ planes, a perfect dislocation of a/2[011] disassociates into two Shockley partials as following:

(1) $$a/2\left[011\right]⟶a/6\left[112\right]+a/6\left[\bar{1}21\right]$$

(2) $$a/2\left[10\bar{1}\right]⟶a/6\left[2\bar{1}\bar{1}\right]+a/6\left[11\bar{2}\right]$$

On further tensile deformation, the interaction of partial dislocations results in the creation of a/6[011] stair-rod dislocation as following:

(3) $$a/6\left[2\bar{1}\bar{1}\right]+a/6\left[\bar{1}21\right]⟶a/6\left[011\right]$$

Since the resultant a/6[011] is not on the active slip planes as shown in Figure 6, it is immobile. The sessile dislocations created like above mechanism are also known as Lomer Cottrell locks. These Lomer Cottrell locks further act to hinder dislocation motion on the slip planes, resulting in enhanced work hardening. In addition to this, Lomer Cottrell locks also stabilize the stacking fault network, which consequently affects the dislocation motion, and also promote nucleation and growth of the HCP phase at the base of stacking faults. They also act to form high density immobile stacking faults. As shown in Figure 6, dislocations are often piled up in front of immobile stacking faults as it is difficult to penetrate them, resulting in additional work hardening.

**Figure 8:**
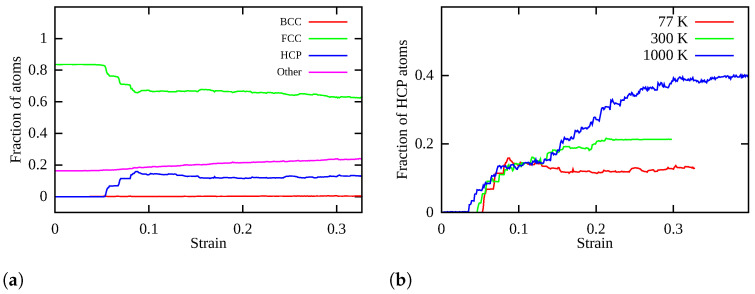
(**a**) Change in fraction of atoms during tensile deformation of CoNiCrFeMn HEA nanowire at 77 K; (**b**) fraction of HCP atoms during tensile deformation of CoNiCrFeMn HEA NW at 77 K, 300 K, and 1000 K.

Figure 8a shows the fraction of atoms in different phases at 77 K with increasing strain. The fraction of FCC atoms firstly decreases in steps at around 0.05 strain and then becomes stagnant through entire tensile deformation. This was also accompanied by a similar increment in fraction of HCP atoms shown by the blue curve in Figure 8a. The number of HCP atoms remains constant before the nucleation of partial dislocations, which on further loading starts increasing. After a saturation of the nucleating dislocations, the number of HCP atoms becomes constant. We further analyze the fraction of atoms in the HCP phase (see Figure 8b) at different temperatures during entire tensile deformation to reveal an FCC-HCP martensitic transformation. The FCC-HCP transformation often takes place with the aid of stacking faults. It is worth noting that HCP phase nucleation begins earlier in the form of partial dislocations at 1000 K than 300 and 77 K. Interestingly, the fraction of HCP atoms at 1000 K is much higher than 77 and 300 K in the later stage of deformation as can be seen in Figure 8b. This confirms a high rate of FCC-HCP martensitic transformation at 1000 K in contrast to 300 and 77 K. Thus, partial dislocation nucleation also promotes temperature-dependent synergistic FCC-HCP martensitic transformation during later stages of tensile deformation as seen in Figure 5b,c. The transformed HCP phase results in the annihilation or unlocking of existing Lomer Cottrell junctions. Due to this, partial dislocations cannot glide through nanowires and fails after necking. This enables the transformation induced plasticity (TRIP) effect at larger strains in the nanowires, which is stronger with increasing temperature.

### 3.3. Superplasticity in Ag NWs and CoNiCrFeMn HEA NWs

**Figure 9:**
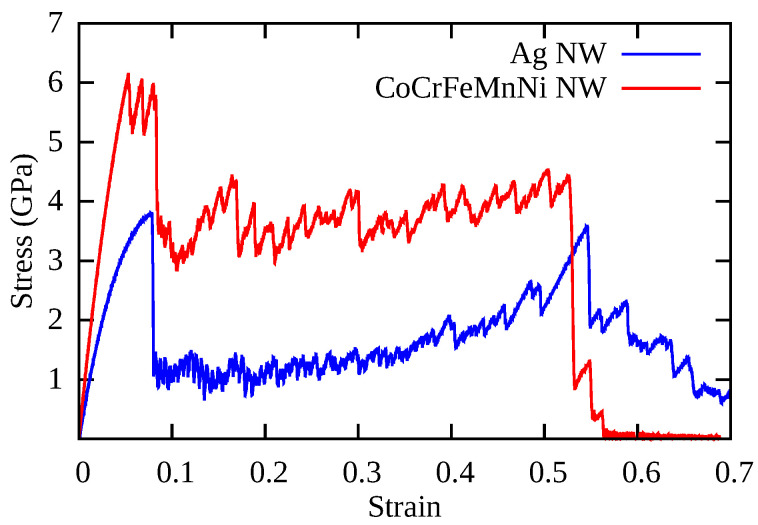
Stress–strain curves of Ag NW and CoNiCrFeMn HEA NW at 77 K. Both the nanowires show excellent superplasticity at cryogenic temperature.

The superplastic behavior of Ag NW and CoNiCrFeMn HEA NW at cryogenic temperature is shown in the Figure 9. In conventional metallic nanowires such as Ag, the operative mechanism for the superplasticity and shape memory effects is deformation twinning, where nucleation and propagation of partial dislocation take place along the NW axis as also shown in Figure 3a. Thus, the newly created twin boundaries maintain the elongation of NW with increased work hardening by delaying the onset of plastic instability of necking. On the other hand, activation of deformation twinning is delayed in CoNiCrFeMn HEA NW, however, superplasticity is still present in CoNiCrFeMn HEA NW at cryogenic temperature. As shown in Figure 9, the stress–strain behavior of CoNiCrFeMn HEA NW shows a fluctuation around the yield point at the stress level of 6 GPa, which represents the simultaneous nucleation of dislocations on parallel slip planes (also see Figure 5a). After certain elongation, a remarkable drop in stress indicates the activation of twinning in the NW. The deformation twins are created by dislocation gliding under uni-axial applied stress which further try to extend through the NW axis and re-orient the area between the twin boundaries. The twin boundaries interact with the dislocations on parallel slip planes and create a complex network of dislocations as shown in Figure 6a. This leads to an ultra high strength, increased work hardening, and delaying the onset of necking resulting in high superplasticity of the CoNiCrFeMn HEA NW at 77 K. Due to lattice distortion and friction between two parallel (111) planes, this effect is limited in HEA NW in comparison to the Ag NW. In addition to this, conventional metallic NWs show a weakening behavior after the yielding, while CoNiCrFeMn NW shows a hardening after the yielding.

To explore the underlying mechanism of superplasticity in the CoNiCrFeMn NWs, the contributions of dislocations on plastic strain at different temperatures are also shown in Figure 7. Dislocation density can be estimated at different stages based on the nucleation and annihilation rate. Interestingly, at 77 K, dislocation density is highest, and at 300 K it is higher than 1000 K. This results in a high yield strength and ductility at 77 and 300 K in comparison to 1000 K as also shown in Figure 2. Additionally, similar to Ag nanowire, twinning induced re-orientation from the (110) phase to (100) phase begins in CoNiCrFeMn HEA NWs at 77 K (see Figure 4). However, the complete re-orientation of the nanowire could not take place (see Figure 5a,b) due to the strong obstacles to the twinning boundary movement from complex dislocation interactions in the form of Lomer Cottrell locks and subsequently from stair rods.

### 3.4. Role of Stacking Fault Energy in the Deformation Mechanism

**Figure A2:**
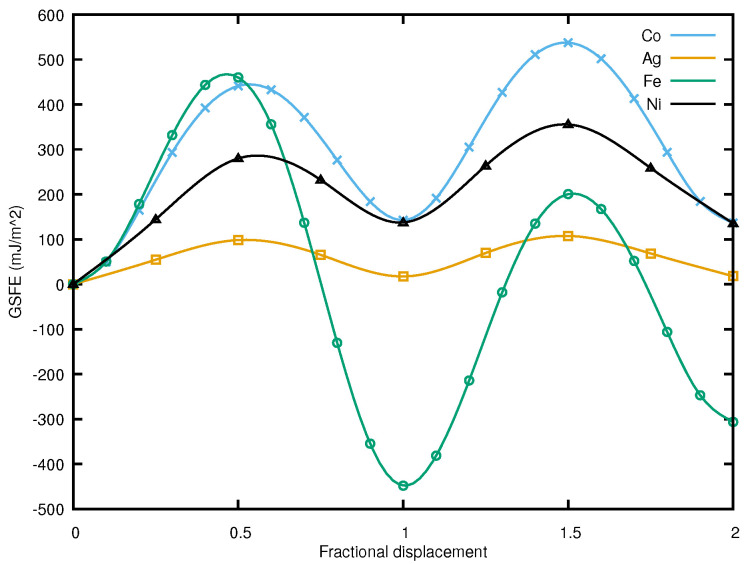
Generalized stacking fault energy curves for the elements.

Surface dislocation nucleation has been identified as a dominant deformation mechanism in NWs contrast to bulk materials. Twinning and dislocation slip are the two competitive mechanisms of deformation in FCC metallic NWs. The former is responsible for large plasticity while the latter results in limited plasticity. The competition between these two mechanisms is dependent on loading direction and material properties such as stacking fault energy. The energy barrier for the twinning is proportional to the change in surface energy. Thus, the transition of deformation modes is dependent on the change in surface energy associated with stacking fault energy. Stacking fault energy (SFE) is an intrinsic parameter which often depends upon composition, alloying elements, and temperature. Materials may exhibit martensite formations at low SFEs, deformation twinning at intermediate SFEs, and dislocation glide at high SFEs. Here, we explore the role of stacking fault energy using generalized stacking fault energy curves of Ag Figure A2 and CoNiCrFeMn obtained from first principle calculations. For CoNiCrFeMn HEA, the values of unstable stacking fault ($\gamma_{usf}$), intrinsic stacking fault ($\gamma_{isf}$), unstable twinning fault ($\gamma_{utf}$) and extrinsic stacking faults ($\gamma_{esf}$) are estimated based on the data from the reference [52]. We further performed the DFT simulations (see Appendix A for details) using the Vienna ab initio simulation package (VASP) [53] to estimate the corresponding values for Ag following the same method in reference [52].

(4) $$\delta_{usf}^{utf}=\gamma_{utf}-\gamma_{usf}$$

The relative barrier height ($\delta_{usf}^{utf}$) determines whether the nucleated partial dislocations can lead to the formation of full dislocations causing dislocation assisted slip to be the plastic deformation mechanism or twinning become the preferred plasticity mechanism. Table 2 shows the positive values of $\delta_{usf}^{utf}$ for both Ag and HEAs. It means at higher values of $\delta_{usf}^{utf}$ dominated mechanism is dislocation slip in case of HEA NWs while twinning is favored in Ag NWs. Another criteria to test the possibility of twinning is suggested by Tadmor and Bernstein [54].

(5) $$\tau_{a}=\left[1.136-0.151\frac{\gamma_{isf}}{\gamma_{usf}}\right]\sqrt{\frac{\gamma_{usf}}{\gamma_{utf}}}$$

where $\tau_{a}$ is a relative measure of the tendency of a FCC metal to undergo deformation twinning. A larger $\tau_{a}$ indicates a greater propensity for twinning. As shown in Table 2, higher $\tau_{a}$ value for Ag indicates a greater tendency of deformation twinning in Ag NW than CoNiCrFeMn HEA NW. The above values are estimations of the deformation behavior at 0 K and are in better agreement with our results at low temperature. At elevated temperatures, there is often an increase in intrinsic stacking fault energy for both Ag and CoNiCrFeMn HEAs as reported in these studies [26,55]. Eventually, the deformation mechanism shifts from deformation twinning to dislocation slip at elevated temperatures.

## 4. Conclusions

In the present work, tensile deformation of Ag NWs and multicomponent HEA CoNiCrFeMn NWs was carried out using molecular dynamics simulations at different temperatures. Based on this study, the following conclusions can be made:

1 It is found that both Ag NWs and HEA NWs show unique superplastic behavior during tensile deformation at 77 K. In HEA NWs, dominant deformation mechanism is TWIP, aided by limited FCC-HCP martensitic transformation during the structural re-orientation, while Ag NW deforms via twinning induced surface re-orientation.
2 At 300 K, during tensile deformation, the motion of partial dislocations is hindered by stacking fault networks resulting in formation of dislocation junctions or Lomer Cottrell locks in HEA nanowire. We speculate that variations in the local chemical composition in HEA nanowires facilitate the formation of Lomer Cottrell locks, which further promotes formation of nucleation sites for the HCP phase transformation. At a later stage of deformation, FCC-HCP martensitic transformation causes unlocking or annihilation of Lomer Cottrell locks. In contrast to deformation at 77 K, martensitic transformation induced plasticity overtakes the twinning induced plasticity in HEA nanowire, while Ag nanowire deforms mainly by dislocation slip with a little contribution from twinning.
3 At 1000 K, FCC-HCP martensitic transformation dominates in HEA NWs, which results in the TRIP effect enabling additional ductility and refractory properties. Similar to 300 K, dislocation junctions or Lomer Cottrell locks are unlocked and annihilated at a later stage of deformation. On the other hand, Ag nanowires fail by dislocation slip.
4 Increasing the deformation temperature of HEA nanowires leads to a decrease in the dislocation density, which is also responsible for shifting the deformation mechanism from TWIP to TRIP at elevated temperatures. With increasing temperature, martensitic transformation dominates as an independent mechanism and results in the annihilation or removal of Lomer Cottrell locks at the later stages of deformation. Thus, the synergistic sequence of plastic deformation mechanisms in HEA NWs at different stages of strain are the source of its excellent combination of strength, ductility, and toughness or damage tolerance which are in better agreement with the existing experimental and simulation studies.

## Figures and Tables

**Table 1:** Crystallographic details of the Ag and CoNiCrFeMn HEA nanowires at different temperatures.

Nanowire	No. of Atoms	Diameter (nm)	Length (nm)	Orientation
Ag	47,250	5	30	X: $\left[\bar{1}10\right]$, Y: [001], Z: [110]
HEA	55,216	5	35	X: $\left[\bar{1}10\right]$, Y: [001], Z: [110]

**Table 2:** Stacking fault energy values obtained from the density functional theory simulations as implemented in VASP [53]. SFE values for CoNiCrFeMn are taken from the reference [52].

Element	a(A)	$\gamma_{\mathrm{usf}}$ (mJ/m^2^)	$\gamma_{\mathrm{isf}}$ (mJ/m^2^)	$\gamma_{\mathrm{utf}}$ (mJ/m^2^)	$\delta_{\mathrm{usf}}^{\mathrm{ut}}$	$\tau_{a}$
Ag	4.15	98.33	17.27	107.46	9.13	1.06
CoNiCrFeMn	3.59	38.50	29.70	56.60	18.10	0.84

**Figure A1:**
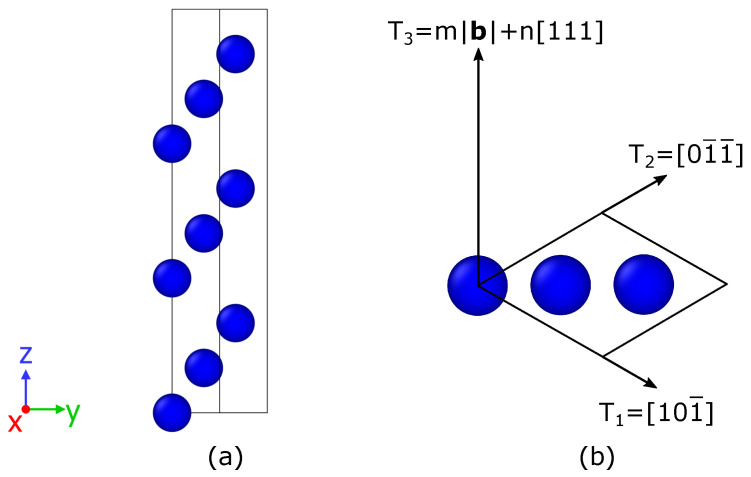
(**a**) Front view of 9 atom supercells used for the SFE calculation. (**b**) Top view of 9 atom hexagonal supercell stacked along (111) fcc planes. Corresponding vectors **T_1_**, **T_2_** and **T_3_** are also shown.

## Data Availability

Video S1 and S2 are openly available in Supplementary Materials by https://drive.google.com/file/d/1OF1A-6BW_k8WI5R5Ew21-di4tVBGRv82/view?usp=sharing.

## Supplementary Materials

The following are available online at https://www.mdpi.com/article/10.3390/nano11082111/s1, Video S1: Animation showing surface re-orientation of CoNiCrFeMn HEA nanowires during uni-axial tensile deformation at 77 K. Video S2: Animation showing complex dislocation activity associated with the interaction of the twin boundary during surface re-orientation of CoNiCrFeMn HEA nanowires during uni-axial tensile deformation at 77 K.

## Abbreviations

The following abbreviations are used in this manuscript:

LC	Lomer Cottrell locks
NW	Nanowire
HEA	High entropy alloy
TWIP	Twinning induced plasticity
TRIP	Transformation induced plasticity

## Appendix A. DFT Simulation Details for the SFE Calculation

We performed the DFT simulations using the Vienna ab initio simulation package (VASP) [53]. Projector augmented wave (PAW) potentials [56], instead of ultra soft pseudopotentials, and the generalized gradient approximation (GGA) [57] are used to enhance the accuracy of the calculations. A periodic supercell, as shown in Figure A1 with no free surfaces and consisting of nine (111) FCC layers with one atom per layer, was used as a reference structure. The upper half of the simulation cell was shifted through one fault vector with magnitude $b_{p}=a_{o}/\sqrt{6}$ along $\left[11\bar{2}\right]$ relative to lower half within a (111) slip plane. After shifting the upper half of the simulation cell, the first maximum along this path is the unstable stacking fault ($\gamma_{usf}$), followed by first minimum as intrinsic stacking fault ($\gamma_{isf}$) at a displacement of $a_{o}/\sqrt{6}$. On further displacement, through one fault vector $b_{p}$ unstable twinning fault ($\gamma_{utf}$) and extrinsic stacking faults ($\gamma_{esf}$) are generated at the upcoming maximum and minimum points, respectively. For these cells, Brillouin zone sampling is performed using 36 × 36 × 6 special k-point mesh with 450 eV energy cutoff, ensuring convergence of energy within 1 meV/atom.
